# Africa’s Digital Health Revolution: The Digital Fit-Viability Model to Move From Innovation to Scaled Implementation

**DOI:** 10.2196/63495

**Published:** 2026-01-14

**Authors:** Afra Jiwa, Antony Ngatia, Karim Benali, Niclas Boehmer, Sangu Delle, Patrick Emedom-Nnamdi, Chris Opoku Fofie, Christine M O’Brien, Tobi Olatunji, Kate Obayabgona, Milind Tambe, Richard Ribon Fletcher, Adeline Adwoa Boatin, Bethany Hedt-Gauthier

**Affiliations:** 1 Program for Global Surgery and Social Change Harvard Medical School Boston, MA United States; 2 Usher Institute University of Edinburgh Edinburgh United Kingdom; 3 Statsspeak Analytics Nairobi Kenya; 4 Global Health and Service Advisory Council Harvard Medical School Boston, MA United States; 5 Hasso Plattner Institute University of Potsdam Postdam Germany; 6 CarePoint Accra Ghana; 7 Department of Biostatistics Harvard T.Chan School of Public Health Boston, MA United States; 8 Ghana Health Service Accra Ghana; 9 Department of Biomedical Engineering Washington University in St. Louis St Louis, MO United States; 10 Intron Health London United Kingdom; 11 Center for Research on Computation and Society Harvard John a. Paulson School of Engineering and Applied Sciences Boston, MA United States; 12 Mechanical Engineering Department Massachusetts Institute of Technology Boston, MA United States; 13 Department of Obstetrics and Gynecology Massachusetts General Hospital Boston, MA United States; 14 Department of Maternal Child Health University of North Carolina Chapel Hill Chapel Hill, NC United States; 15 Department of Global Health and Social Medicine Harvard Medical School Boston, MA United States

**Keywords:** digital health, Africa, digital health solutions, digital technologies, health care access, fit/viability model

## Abstract

Digital innovations hold immense potential to transform health care delivery, particularly in sub-Saharan Africa, where financial, geographical, and infrastructural constraints continue to hinder progress toward universal health care delivery. Although a growing health tech sector offers creative solutions, few digital health interventions reach scaled implementation. In this paper, we present the digital fit/viability model—an adapted determinant framework to describe facilitators and barriers to moving from digital tools to integrated digital health implementation. We then use this model to describe the specific challenges and recommended solutions when developing digital health tools for health systems in sub-Saharan Africa.

## Introduction

Digital tools can improve health care delivery and enhance health outcomes by addressing long-standing challenges in health care access, quality, and efficiency [1]. This potential is particularly salient in sub-Saharan Africa, where persistent barriers to health care, including inadequate infrastructure, workforce shortages, and resource limitations, could be alleviated or entirely bypassed with digital health interventions [2,3]. There are notable examples of scaled digital health interventions in sub-Saharan Africa. For example, District Health Information Software 2, a digital informatics platform operating in 40 countries, facilitates electronic data entry at health facilities, making information available for surveillance and program monitoring in near real time [4]. Zipline offers a digital platform for immediate order and drone-led distribution of health products in Rwanda [5]. In Kenya, M-TIBA allows users to access their health insurance through a mobile payment technology called m-pesa [6]. However, despite some successes, the digital health field suffers from “pilotitis,” where the vast majority of digital interventions do not move beyond the pilot phase [7].

Over the last decade, the World Health Organization (WHO) and the World Bank have issued several policy documents outlining priority areas for digital interventions, as well as identifying systems gaps that need to be addressed for digital health interventions to achieve their full potential [8-11]. However, these documents do not systematically specify the facilitators and barriers that affect the progression of a specific digital health tool from concept to implementation. Drawing from our experience in developing, researching, and implementing digital health interventions in sub-Saharan Africa, we introduce the digital fit/viability model (dFVM) for health. This model adapts the fit/viability model developed by Liang et al [12] for the use of mobile technology in business and is an example of an implementation science determinant framework to describe the facilitators and barriers that influence implementation outcomes. In this paper, we explain how this model can be adapted for digital health interventions and use dFVM to illustrate challenges specific to sub-Saharan Africa as well as the potential solutions.

## Positionality Statement

This work is the result of a 2-day convening of a multidisciplinary group of clinicians, researchers, technologists, entrepreneurs, policymakers, and health delivery service providers. The group included individuals with experience in Ghana, Kenya, Nigeria, Rwanda, Tanzania, Uganda, United States, and United Kingdom, and spanned different age groups, ethnicities, genders, and professional experience; all had experience of working within the African digital health ecosystem. The group held the shared perspective that digital tools have the potential to transform health care in sub-Saharan Africa. From this perspective, we discussed challenges to the implementation of digital innovations in these contexts and strategies to develop, evaluate, and scale context-appropriate and sustainable digital health solutions.

## Digital Tools, Interventions, and Implementation

Here, we define digital health tools, interventions, and implementation to clarify the role of dFVM. Digital health tools encompass a variety of technology-enabled products and services developed to improve health care services [13]. Broadly, digital health tools have been categorized into virtual interactions (such as platforms for teleconsultations), paperless data (such as electronic health records), patient self-care (such as mobile apps to support chronic disease management), patient self-service (such as e-booking platforms), decision intelligence systems (such as hospital patient flow management systems), and workflow automation (such as medical equipment tracking systems using radiofrequency identification) [13]. Digital health interventions refer to the services, practices, and strategies that utilize digital health tools. For example, Zipline is an intervention that engages a cluster of digital tools—drones, online ordering systems, etc—to facilitate timely order-to-delivery of health products. Finally, digital health implementation involves integrating a digital health intervention into broader health care delivery. For instance, the successful deployment of Zipline is embedded within the larger health care framework, where the ordered health products are identified as part of patient care and delivered accordingly, outside of the Zipline system itself. Despite a surge in digital health tools over the past decade, few have successfully been integrated into interventions, and even fewer digital health interventions have achieved widespread, sustained implementation into health care systems, particularly in sub-Saharan Africa [1].

## The Fit/Viability Model

**Figure 1:**
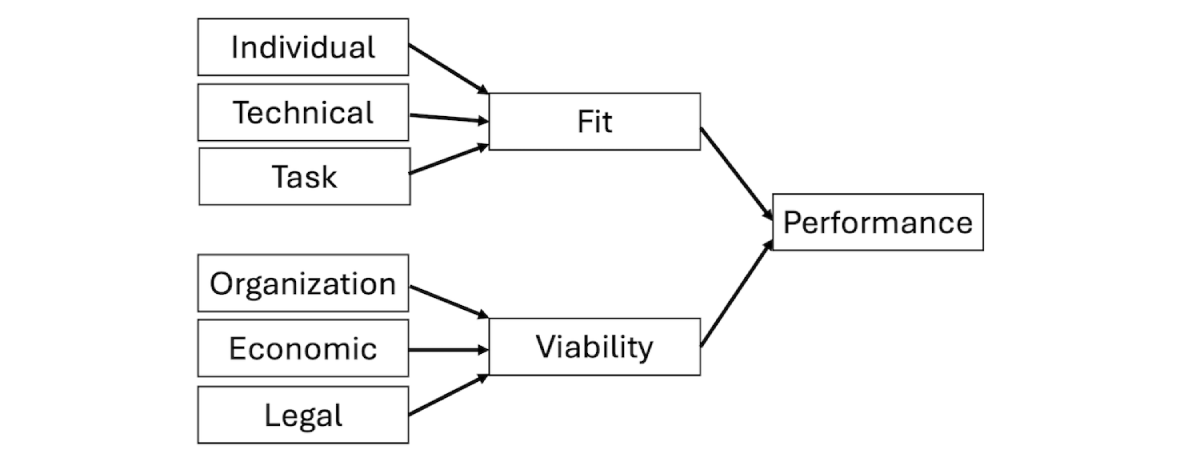
The digital fit/viability model (adapted from Liang et al [12], which is published under Creative Commons Attribution 4.0 International License [15]).

Liang et al [12] developed the fit/viability model in 2007 to support the adoption of new technology in the business sector [14]. In this original framework, “fit” evaluates how well a technology aligns with its intended environment, while “viability” assesses whether the technology can be feasibly implemented given the organization’s constraints. Both fit and viability are essential for a technology to succeed at scale within a business. These overarching “fit” and “viability” questions have been valuable for our team when developing digital health tools as part of complex interventions in sub-Saharan Africa. In the next section, we explain how we adapted Liang et al’s [12] framework and incorporated additional subdomains to create dFVM for health (Figure 1; [15]).

## dFVM Concept

### Defining the “Fit” of Digital Health Tools

Similar to that defined by Liang et al [12], we define “fit” as the appropriateness of a digital health tool in a specific setting as part of a specific intervention. Generally, we are asking questions such as “Who will be using this digital health tool?”, “What is this digital health tool designed to do?”, and “Does this digital health tool align with the organizations aims?” (Figure 1). These questions are best addressed in the early design and conception stages. Although these fit concepts are important for a digital health tool in any context, the particular challenges in sub-Saharan Africa require deeper exploration of these questions.

### Individual Fit

Individual fit, which was not included in the original fit/viability model, assesses whether the digital health tool is appropriate for those who engage with the tool. This category emphasizes that technologies should be designed with end users in mind.

#### Challenge

In sub-Saharan Africa, one particular challenge for individual fit is the varied levels of digital literacy [16,17] and access to digital platforms [9,18]. For example, a digital intervention that requires a patient to have a smartphone to engage with an app may fail either because the patient does not own a phone and/or because the patient is not comfortable navigating a digital App.

#### Solution

As a first step, anyone developing a digital tool should conduct end user digital accessibility assessments, including assessing digital literacy, device ownership, and access to other system requirements such as cell networks or electricity. There are numerous tools to assess digital literacy [14], but few are designed specifically for a sub-Saharan African context. One alternative is to outline the gaps in device ownership or digital literacy that must be addressed when deploying the digital health intervention so that anyone adopting a digital health tool understands the pathway to ensure individual fit.

### Technical Fit

Technical fit assesses whether the technological infrastructure exists to support the digital health tool. This domain was not included in the original fit/viability model developed by Liang et al [12], which focused on deploying e-commerce tools in high-income country contexts—settings already primed for new technologies. In our iteration, we consider the potential mismatch between the infrastructure in which a tool was designed and the setting in which it will be deployed.

#### Challenge

In many sub-Saharan African settings, foundational infrastructure such as electricity and internet remains unreliable, and hardware needs such as hardware durability, power consumption, and maintenance are often overlooked. Tools developed for high-income country contexts may rely on stable cloud access, regular software updates, or consumables that are expensive or difficult to source in sub-Saharan Africa. As a result, imported technologies often end up in “equipment graveyards,” unused due to incompatibility with local systems, lack of technical support, or excessive training requirements [19-22].

#### Solution

The first question should often be “Are there nondigital solutions that are equally suited for this task?”, ensuring that implementers are not missing more accessible, viable, and equally impactful solutions. When digital health interventions offer clear advantages, developers must assess infrastructure needs early and design for low-resource environments, considering offline functionality, portability, power efficiency, and local repair capacity. Nationally, more advocacy is needed to support initiatives that expand digital infrastructure. Projects like Health Connect Africa [23], a partnership between Centre for Disease Control Africa and Global System for Mobile Communications Association, which aims to connect 10,000 health care facilities to the internet by 2030, and Power Africa [24], which focuses on electrifying health facilities, are examples of essential infrastructure-building efforts that make digital health tools viable at scale.

### Task Fit

We expand Liang et al’s [12] considerations of task fit to assess whether a digital health tool is able to complete the task for which it is designed. A tool may be well matched to the infrastructure and the user but still fail to achieve the task for which it is intended.

#### Challenge

In the digital health field, we often start with tools developed for high-resource environments and adapt these tools to accomplish specific tasks in sub-Saharan Africa [25], which can lead to numerous problems. One notable example is for digital tools that engage artificial intelligence. These often do not include data representative of individuals in sub-Saharan Africa and can have high bias and low validity when implemented in those settings [26].

#### Solution

First, we must center health program implementers and policymakers to identify the priority gaps we are addressing through digital health interventions. Once we have these priority areas, we must move beyond simply adopting tools and instead validate them through dedicated studies, be willing to adapt or retrain them for context, and, when necessary, design tools from scratch. For AI-enabled digital tools, better and more accessible datasets are needed on the African continent. Although funding and data formatting have been obstacles, recent initiatives such as the National Institutes of Health–funded DS-i Africa [27] along with Masakhane [28], the Lacuna Fund, and Zindi [29], are actively creating shared open repositories. These platforms will accelerate health care innovation by providing representative, high-quality data that directly addresses the bias and validity issues of the existing tools.

### Defining the "Viability" of Digital Health Tools

Liang et al [12] considered viability as the organization’s economic, technical, and social readiness to adopt a digital tool. We have expanded this to also include legal and ethical considerations that present significant challenges when developing digital tools for interventions and implementation in sub-Saharan Africa. The viability domain considers “Can this tool or intervention thrive in the ecosystem into which it is being deployed?”, “Does this tool or intervention have the legal and financial backing to be sustainable?”, and “Is the technical infrastructure available to support this tool?” Although fit questions should be addressed early, viability questions are addressed as the digital tool is integrated into interventions and implemented on small or broad scales.

### Organization Viability

In the original model, organization viability describes factors such as management support, digital literacy of team members, user competence, and experience [12]. We have shifted several of these individual features such as digital literacy and user competence to “fit” since these must be addressed early as the digital tool is being developed. We have expanded the management support to include plausible integration of the tool into program implementation structures. Adopting technologies that are well matched to their organizational environment can promote uptake and sustainability.

#### Challenge

Organizational viability considers whether health systems and institutions have the leadership, governance, and operational capacity to sustain digital health interventions. In sub-Saharan Africa, frequent staff turnover and fragmented leadership structures often undermine long-term adoption [30]. Ministries of health may have alternative health priorities or lack the resources to integrate new tools across departments and levels of care. These factors mean that even technically sound and contextually appropriate interventions may struggle to move beyond pilot projects if organizational systems are not aligned to support them.

#### Solution

Early involvement of multidisciplinary teams is key to assessing feasibility and organizational readiness. Usability testing can help establish workflows, training, and tools that support smooth adoption. For developers, designing solutions that integrate easily with existing infrastructure and that can be bundled with current systems promotes interoperability and long-term alignment. Engaging leadership early and ensuring interventions reflect national health priorities helps secure ownership, buy-in, and resource allocation, thereby reducing the risk of tools operating in isolation from the systems they aim to strengthen.

### Economic Viability

In the study of Liang et al [12], economic viability encompasses a cost-effectiveness and cost-benefit analysis. We extend this definition to ask whether a digital health intervention can be sustained financially, including through Ministries of Health and Finance, with the support of philanthropy or multilateral organizations or through the private sector. 

#### Challenge

The digital health sector in sub-Saharan Africa is a patchwork of solutions, with many promising digital health interventions failing to achieve scale due to a lack of coordinated national strategies or shared infrastructure [31]. This decentralized approach, often led by individual clinicians, researchers, and small nongovernmental organizations, limits the ability to benefit from economies of scale [32]. This is compounded by persistent infrastructural barriers like high data costs, unreliable electricity, and poor connectivity as well as inconsistent funding avenues [33]. These issues place a disproportionate financial burden on smaller organizations and new companies. The high cost of entry and limited seed funding make it difficult for promising early-stage innovations to gain traction, leading to isolated successes rather than widespread impact.

#### Solution

To ensure the continued funding of digital health interventions, a combination of coordinated investment and new funding models are needed. Blended financing, which combines public funds, donor contributions, and socially responsible investment, can help bridge the gap and move interventions from pilot to scale [34]. Health systems–level investment into basic infrastructure and platform will make any single digital health intervention more economical and cost-beneficial [9].

### Legal Viability

Legal viability refers to the presence of clear, enforceable policies that govern the implementation of digital health technologies, with a focus on data privacy, security, and system interoperability. Although this was not included in Liang et al’s [12] model, in our experience, this has been a major challenge and often missed step for digital health interventions in sub-Saharan Africa.

#### Challenge 

National and regional policies for digital health interventions are nascent in sub-Saharan Africa. Without clear regulations on data privacy, security, and interoperability, implementers face an uncertain and rapidly changing regulatory landscape [35-37]. This lack of maturity in frameworks creates significant uncertainty for both innovators and investors, making it difficult to establish a strong foundation for technological advancement [35].

#### Solution

Strengthening legal viability requires investing in national and regional regulatory capacity and developing robust data governance systems. This includes improving regulations to ensure trustworthiness while still supporting innovation [38]. Harmonizing standards across borders will facilitate the ability to share and use tools beyond the specific contexts for which they were developed [36].

## dFVM Application

Table 1 shows a summary of the application of dFVM to identify the challenges and possible solutions for implementing digital health interventions in sub-Saharan Africa.

**Table 1:** Applying dFVM^a^ to identify the challenges and possible solutions for implementing digital health interventions in sub-Saharan Africa.

Domains, subdomains of dFVM			Challenges and solutions identified when applying dFVM to sub-Saharan Africa		
			Challenges		Solutions
**Fit**					
	Individual	Limited digital literacy and access to digital platforms among potential end users.		Conduct end user digital assessments, including digital literacy and device ownership assessments prior to tool development. Build digital skills and awareness as part of intervention development.	
	Technical	Unreliable electricity, frequent power outages, poor internet connectivity, high cost of mobile internet data.		Consider nondigital alternatives. Assess infrastructural capacity early and develop tools that comply to that infrastructure. Invest in foundational infrastructure to connect facilities, health workers, and patients.	
	Task	Imported technologies may not align with the actual tasks or workflows in health systems. Tools often assume task environments similar to high-income countries. Artificial intelligence tools can also perform poorly due to algorithmic bias.		Address priority areas identified by health practitioners and policymakers in the local context. Ensure co-design with frontline health workers to reflect actual care tasks and workflows. Test, validate, and adapt digital health tools as needed. Build Africa-specific data repositories.	
**Viability**					
	Organizational	Fragmented leadership, staff turnover, and organizational priorities, which differ from the tools that are developed.		Bundle tools with existing infrastructure, promoting interoperability and integration; engage leadership early; and promote system-level alignment and ownership.	
	Economic	Insufficient funding and lack of sustainable financing mechanisms leads to premature abandonment of promising initiatives or poor scaling of established interventions.		Invest in national digital health platforms, which will reduce the per-tool costs. Prioritize national funding models, complemented by blended public-private financing.	
	Legal	Lack of clear policies and regulations regarding data privacy, security, and interoperability.		Develop robust data governance and digital regulations for countries. Seek, where possible, cross-continent regulations to facilitate portability.	

^a^dFVM: digital fit/viability model.

## Discussion

We developed dFVM to help individuals designing digital health tools and interventions, particularly innovators working in sub-Saharan Africa, think more critically about the development of these tools and interventions with the goal of successful digital health implementation. We have identified 3 other determinant frameworks relevant to digital health interventions, namely, the Train, Restructure, Incentivize, Mandate, Integrate (TRIMI) framework [16]; a modified version of the Consolidated Framework for Implementation Research (mCFIR) for mobile health tools [17]; and a third developed by Olu et al [3]. Each of these frameworks offers a different vantage to consider when developing digital health tools and interventions. For this paper, we adapted the fit/viability model because this most closely mirrored how we approach our digital tool development—asking questions about whether a digital health tool will fit a specific task, for a specific user, in a specific infrastructure, and whether a digital health tool achieves the organizational integration, financing, and legal compliance needed to be viable.

Although applicable to any digital health tool, we made sure that dFVM addresses the unique obstacles we have encountered in sub-Saharan Africa. Throughout our description of dFVM, we have highlighted these challenges and offered solutions. Broadly, these solutions fall into 2 buckets. First, what is the responsibility of the individuals developing the tools and interventions? These individuals must design for context and, through the deployment of digital health interventions, build institutional capacity for digital health interventions more broadly. Second, in the context of sub-Saharan Africa, governments play a crucial role, particularly in ensuring the viability of digital health interventions. Investing in digital infrastructure provides a foundation for sustaining various tools and reduces per-tool costs. Moreover, establishing robust legal frameworks, including cross-national agreements, ensures compliance and demonstrates government commitment as partners alongside private and public technology providers.

dFVM offers high-level guidance for individuals developing digital health tools and interventions to ensure their fit and viability within health systems. However, we recognize that moving from idea-to-implementation requires more detailed steps and involves multidisciplinary teams capable of navigating these complexities. As a next step, we are expanding dFVM and integrating it with other digital health implementation science frameworks to develop a step-by-step roadmap to guide teams throughout the development process.

### Conclusion

We believe that digital health interventions have great potential to transform health care delivery and improve outcomes worldwide. However, successfully implementing these interventions in sub-Saharan Africa requires careful attention to their fit and viability within local contexts. dFVM maps challenges across both digital health design and deployment environments and links these challenges to practical solutions drawn from lived experience. Although digital health tool developers are responsible for addressing these considerations, governments in sub-Saharan Africa play a crucial role in ensuring that the necessary organizational and legal frameworks are established to support the adoption of digital health interventions.

## Abbreviations

dFVM: digital fit/viability model
mCFIR: modified version of the Consolidated Framework for Implementation Research
TRIMI: Train, Restructure, Incentivize, Mandate, Integrate
WHO: World Health Organization
